# Knockdown of estrogen receptor β increases proliferation and affects the transcriptome of endometrial adenocarcinoma cells

**DOI:** 10.1186/s12885-019-5928-2

**Published:** 2019-07-29

**Authors:** Oliver Treeck, Elisabeth Diepolder, Maciej Skrzypczak, Susanne Schüler-Toprak, Olaf Ortmann

**Affiliations:** 1 Department of Obstetrics and Gynecology, University Medical Center Regensburg, Landshuter Str. 65, 93053 Regensburg, Germany; 2 Second Department of Gynecology, Medical University of Lublin, Lublin, Poland

**Keywords:** Estrogen receptor β, siRNA, Endometrial cancer, HEC-1A, RL95/2, Transcriptome analysis

## Abstract

**Background:**

Estrogen receptor β (ERβ) has been repeatedly suggested to play important roles in hormone-dependent cancer like in tumors of the breast, ovary or prostate. In this study, we intended to further elucidate its role in endometrial cancer.

**Methods:**

For this purpose, we knocked down ERβ expression in two endometrial cancer cell lines, the ERα-negative/ERβ-positive line HEC-1A and the ERα/β-positive cell line RL95/2, by means of siRNA transfection. Cell proliferation after transfection was assessed using the fluorescent CTB Assay (Promega). In order to elucidate possible molecular mechanisms which might underlie the effect on proliferation, we performed transcriptome analyses by means of human Affymetrix Human Gene Chip 2.0. Additionally, we treated the employed cell lines with different ERβ modulators to examine their effect on proliferation.

**Results:**

siRNA-mediated knockdown of ERβ significantly increased proliferation of both endometrial cancer cell lines. In HEC-1A cells, proliferation was significantly increased 4, 5 and 6 days after transfection, with a maximum of about 1.7-fold (*p* < 0.05) on day 6. Endometrial RL95/2 cells with an ERβ knockdown exhibited a clearly enhanced proliferation on day 3 and days 4 to 8, when even 2.4-fold higher numbers of viable cells were detected (*p* < 0.01). Transcriptome analysis revealed that this was accompanied by increased expression of several genes being known to be upregulated in cancer, including proliferation-associated genes and oncogenes, and by repression of genes associated with differentiation, apoptosis or growth inhibition. Corroborating the observed knockdown effects, treatment with the ERβ antagonists PHTTP and (R, R) THC was also able to induce proliferation of both cell lines.

**Conclusions:**

Our data clearly support the putative role of ERβ as tumor suppressor in endometrium as previously suggested in studies on other tissues and encourage further studies to find out to what extent this molecule might be a potential therapy target in this cancer entity.

**Electronic supplementary material:**

The online version of this article (10.1186/s12885-019-5928-2) contains supplementary material, which is available to authorized users.

## Background

Endometrial cancer is the most common malignancy of the genital tract among women in western countries. It is the third most common cause of gynecologic cancer death behind ovarian and cervical cancer [1]. Type 1 endometrial cancer, also known as endometrioid endometrial carcinoma, is the most frequent subgroup (80%), and shows estrogen and progesterone receptor expression. It emerges from hyperplastic endometrial tissue and often is characterized by PTEN loss in 37–61% of all cases. Type I tumors are typically well differentiated and have a good prognosis, with a low rate of recurrence of approximately 20% [2]. Type 2 endometrial cancer, including serous (10–15% of all cases) and clear cell carcinoma (about 5%) [3] is characterized by frequent loss of E-Cadherin or by HER2 overexpression [4]. For regulation of normal endometrial function, expression of sexual steroid hormone receptors like estrogen receptors (ER) α and β and progesterone receptor (PR) plays an important role. Alterations of ER and PR expression, as well as the ERα/β ratio, have been suggested to be involved in the development of endometrial cancer and loss of these receptors during carcinogenesis has been reported to be associated with an aggressive clinical course and a poorer survival of endometrial cancer patients [5, 6].

Whereas ERα is thought to primarily mediate the proliferative effect of estrogens in endometrial tissue, the complete function of ERβ, which is known to partially antagonize ERα action, is still not fully understood. With regard to endometrial cancer, the role of this receptor is still controversial. Whereas various studies report downregulation of ERβ in endometrial cancer [7–9], others observed increased expression of this receptor in endometrial tumor tissue or its association with disease progression [10–12]. From other hormone dependent tissues like the breast, ERβ is known to exert inhibitory effects on proliferation and invasion, both dependent and independent from presence of ERα [13–15]. The growth inhibitory action of ERβ and the observed decline of ERβ expression during carcinogenesis suggested that this receptor acts as a tumor suppressor in various tissue types [16–18].

The aim of our study was to examine to what extent ERβ might exert tumor suppressor functions in endometrial cancer cells. For this purpose, we knocked down its gene expression by means of RNAi in HEC-1A (ERα^−^/ERβ^+^) and RL95/2 (ERα^+^/ERβ^+^) cells, treated them with specific agonists and antagonists and examined changes of cellular proliferation and the transcriptome of both cell lines (Affymetrix GeneChips).

## Methods

### Materials

DMEM/F12 culture medium, FBS, sodium pyruvate, insulin, L-glutamine and Accutase were obtained from Sigma-Aldrich (Munich, Germany). HEC-1A (ATCC® HTB-112) and RL95–2 (ATCC® CRL-1671) endometrial adenocarcinoma cells were obtained from American Type Culture Collection (Manassas, USA) and were directly propagated for the experiments performed. Affinity Script Multi Temperature cDNA Synthesis Kit was from Agilent (Santa Clara, USA). RNeasy Mini Kit, RNase Free DNase Set and Quantitect SYBR Green PCR Kit were obtained from Qiagen (Hilden, Germany). PCR primers were synthesized at Eurofins (Germany). Transfectin reagent was obtained from BioRad (Hercules, USA). OptiMEM medium were purchased at Invitrogen (Karlsruhe, Germany). ESR2 siRNAs were from Thermo Fisher (Woodward, USA).

### Cell culture and siRNA transfection

HEC-1A and RL95/2 endometrial adenocarcinoma cells were obtained from ATCC and cultured in DMEM-F12 containing 10% FCS at 5% CO_2_ and 37 °C in a humidified incubator. All experiments were performed shortly after purchase of the cell lines. For transfection, 4 × 10^5^ cells per well of a 6-well dish were seeded in DMEM/F12 containing 10% FCS. After 24 h, cells were transfected with 60 nM siRNA in OptiMEM reduced serum medium using 8 μl of Transfectin reagent (BioRad, Hercules, USA). For knockdown of ERβ expression, we used an equimolar mixture of three different pre-designed Silencer siRNAs (20 nM each) (IDs: 145909, 145910 and 145911), Thermo Fisher, Waltham, USA), targeting different regions of ESR2 gene. As a negative control siRNA verified not to interact with any human RNA, 60 nM of the Silencer Negative Control #1 siRNA (AM4611, Thermo Fisher) was used. Three days after siRNA treatment, cells were harvested and total RNA and protein was isolated.

### RNA isolation and qPCR

Total RNA from the cell lines was isolated using RNeasy Micro Kit (Qiagen) according to manufacturer’s protocol. RNA analysis was performed as described earlier [15]. In brief, after reverse transcription, mRNA levels were determined by qPCR. For this purpose, 4 μl of cDNA were amplified using LightCycler® FastStart DNA Master^PLUS^ SYBR Green I (Roche Diagnostics GmbH, Mannheim, Germany) and 5 mM of each intron-spanning primer (Additional file 1: Table S1). qPCRs were carried out in a LightCycler® 2.0 Instrument (Roche, Mannheim, Germany). A β-actin fragment was amplified in parallel in each experiment as reference using intron-spanning PCR primers. RT-qPCR data were then analyzed using the comparative ΔΔC_T_ method [19, 20].

### Western blot analysis

For protein preparation, 72 h after transfection, cells were lysed in RIPA buffer as described earlier [15]. Aliquots of cell lysate containing 10 μg of protein were resolved by 10% (w/v) SDS–polyacrylamide gel electrophoresis, followed by electrotransfer to a PVDF hybond (Amersham, UK) membrane. Immunodetection was carried out using monoclonal ESR2 antibody PPZ0506 (1:500), (#MA5–24807, Thermo Fisher), and β-actin antibody (1:500) (ab8226, Abcam). The secondary antibody was an anti-mouse horseradish peroxidase conjugated secondary antibody (1:20000). Signals were detected using chemiluminescence (ECL) (Amersham, Buckinghamshire, UK). The Western blot results from three independent protein isolations were analysed densitometrically by means of ImageJ software (NIH, USA) and expressed in percentage of cell transfected with negative control siRNA.

### Cell proliferation assays

Parallel to qPCR-based verification that siRNA-triggered knockdown of ERβ was more than 70% effective, the transfected cells, each 100 μl per well, were seeded in triplicates in a 96-well chamber in DMEM-F12 containing 10% FCS. At days 0, 3, 4, 5, 6, 7 and 8, relative numbers of viable cells were measured in comparison to cells treated with negative control siRNA using the fluorimetrical, resazurin-based Cell Titer Blue (CTB) assay (Promega, USA), according to the manufacturer’s instructions and as described earlier [21]. Cell growth was expressed either as percentage of day 0 or as percentage of the solvent controls.

### GeneChip™ microarray assay

For transcriptome analyses using GeneChip Human Gene 2.0 ST Arrays (Affymetrix), RNA from both cell lines was isolated 72 h after siRNA transfection by means of the RNeasy Micro Kit (Qiagen) according to manufacturer’s protocol. Sample preparation for microarray hybridization was carried out as described in the Affymetrix GeneChip® Whole Transcript (WT) Sense Target Labelling Assay manual (Affymetrix, Inc., Santa Clara, CA, USA). In brief, 300 ng of total RNA were used to generate double-stranded cDNA. First, cRNA was synthesized (WT cDNA Synthesis and Amplification Kit, Affymetrix), purified and reverse transcribed into single-stranded (ss) DNA. Purified ssDNA was then fragmented and labelled with biotin (WT Terminal Labelling Kit, Affymetrix). Finally, 2.3 μg DNA were hybridized to GeneChip Human Gene 2.0 ST Arrays (Affymetrix) for 16 h at 45 °C in a rotating chamber. Hybridized arrays were washed and stained in the Affymetrix Washing Station FS450 using Hyb, Wash & Stain Kit (Affymetrix), and the fluorescent signals were measured in the Affymetrix GeneChip® Scanner 3000-7G. Sample processing was performed at the Affymetrix Service Provider and Core Facility, “KFB - Center of Excellence for Fluorescent Bioanalytics” (Regensburg, Germany; http://www.kfb-regensburg.de).

### Microarray data analysis

Using the RMA algorithm in the Affymetrix GeneChip Expression Console Software, summarized probe signals were created. They were exported to Microsoft Excel, and average signal values, comparison fold changes and significance *P* values were calculated. Probe sets with a fold change above 2.0 fold and a student’s t test *p* value lower than 0.05 were considered as significantly regulated.

### Statistical analysis

Statistical analysis of gene expression was performed by means of student’s t-test. For statistics, we used Graph Pad Prism Version 7.04 Software (Graph Pad, San Diego, USA). Statistical significance was stated in case of *p*-values being lower than 0.05.

## Results

### Knockdown of ERβ increased proliferation of endometrial HEC-1A and RL95/2 cancer cells

**Fig. 1:**
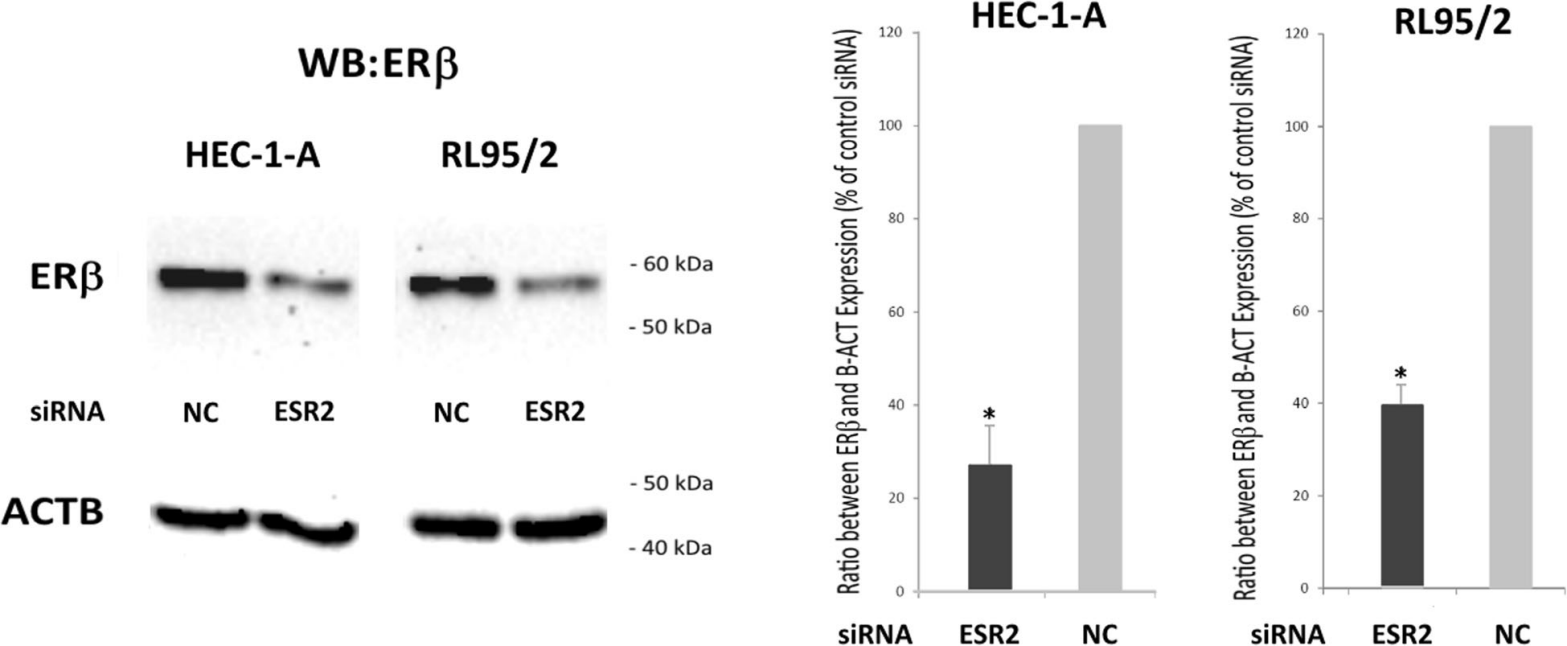
Verification of siRNA-mediated ERβ knockdown in the indicated endometrial cancer cell lines by means of Western blot analysis. Left Panel: Representative Western blot. Right Panel: Diagram showing the mean band intensities from 3 experiments normalized with the housekeeping gene ACTB. The indicated cell lines were transfected either with ESR2 siRNA or negative control (NC) siRNA. *p < 0.01 vs. negative control (*n* = 3)

**Fig. 2:**
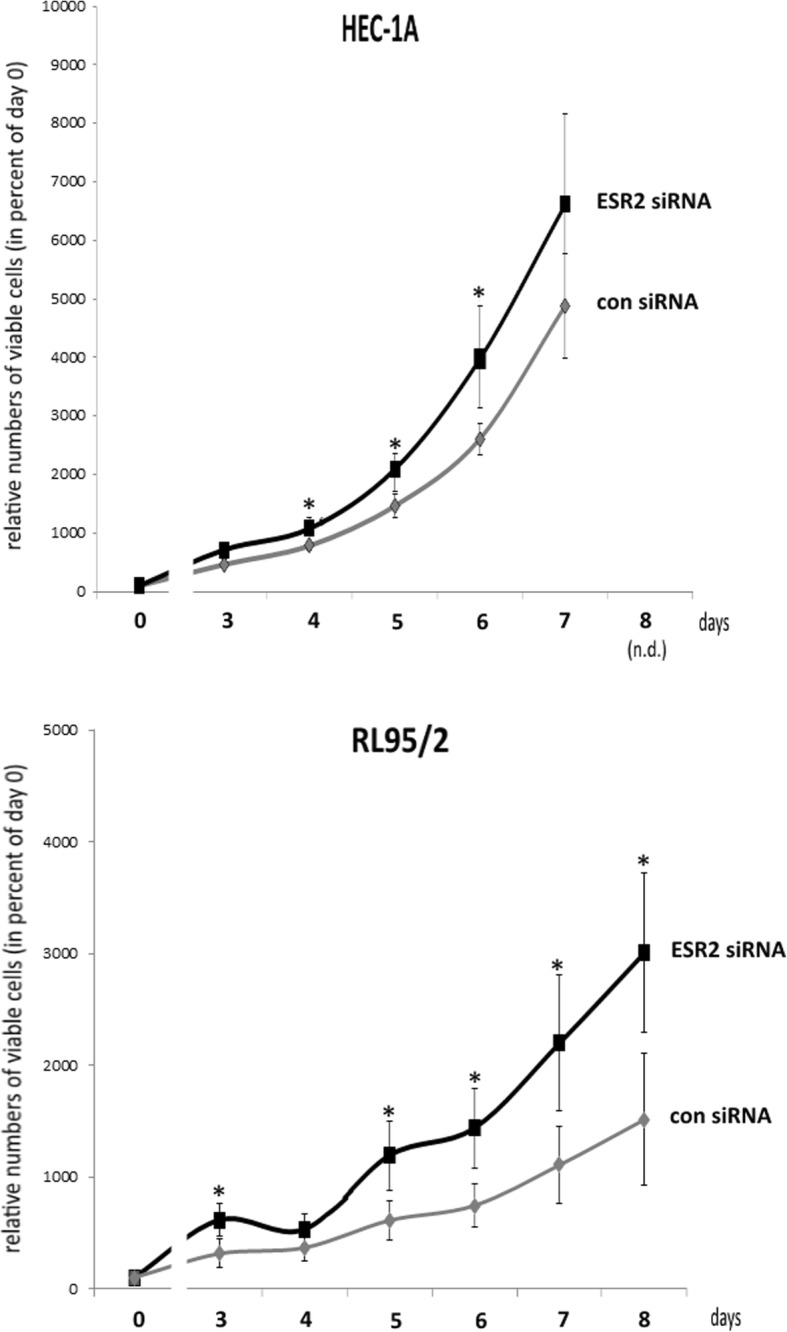
Proliferation of the indicated endometrial cancer cell lines after transfection with ESR2- or negative control siRNA. Measured were the relative numbers of viable cells in percent of day 0, using the fluorometric CTB assay (Promega) *p < 0.05 vs. negative control (*n* = 4)

For our experiments, we employed the well characterized cell line HEC-1A, known to be E2-unresponsive due to lack of ERα expression, and the hormone-responsive and ERα/β positive cell line RL95/2 (see cell line characteristics at ATCC, American Type Culture Collection). To initially confirm ERα status of both cell lines, we performed RT-qPCR experiments demonstrating strong expression of ESR1 gene in RL-95/2, but not in HEC-1A cells as expected (data not shown). To examine the role of ERβ in proliferation of HEC-1A and RL95/2 endometrial cancer cells, both lines were transfected with ESR2-specific siRNA and a negative control siRNA. Efficacy of the knockdown was confirmed by Western blot analysis (Fig. 1) and by RT-qPCR (data not shown). In HEC-1A cells, ERβ protein levels were found to be reduced to 28.7% after ESR2 siRNA transfection, whereas in RL95/2 cells, protein levels of this gene were reduced to 39.5%. Both endometrial cancer cell lines transfected with ESR2 siRNA exhibited an enhanced proliferation (Fig. 2). In HEC-1A cells, a statistically significant increase of proliferation was detected on days 4, 5 and 6 after transfection, with a maximum 1.7-fold increase measured on day 6 (*p* < 0.05). Endometrial RL95/2 cells with an ERβ knockdown exhibited a clearly enhanced proliferation on day 3 and days 5 to 8, when even 2.4-fold higher numbers of viable cells were detected (*p* < 0.01).

### Transcriptome changes after knockdown of ERβ by means of siRNA transfection

**Fig. 3:**
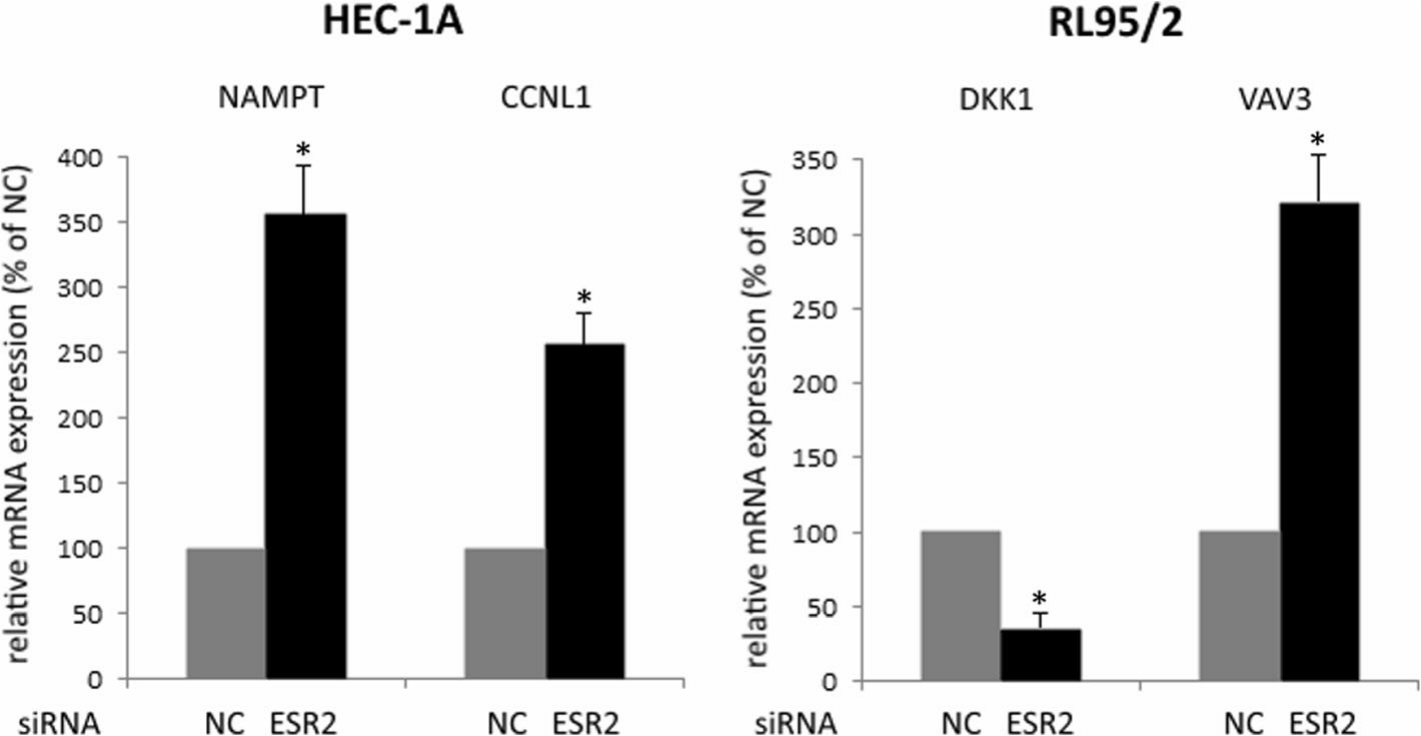
DNA microarray data verification by means of RT-qPCR analysis of the indicated genes. Shown is the relative gene expression 96 h after transfection with negative control (NC) or ESR2 siRNA, expressed in percentage of their mRNA levels in NC-transfected cells. RT-qPCR data were processed using the ΔΔC(T) method (*n* = 3)

**Fig. 4:**
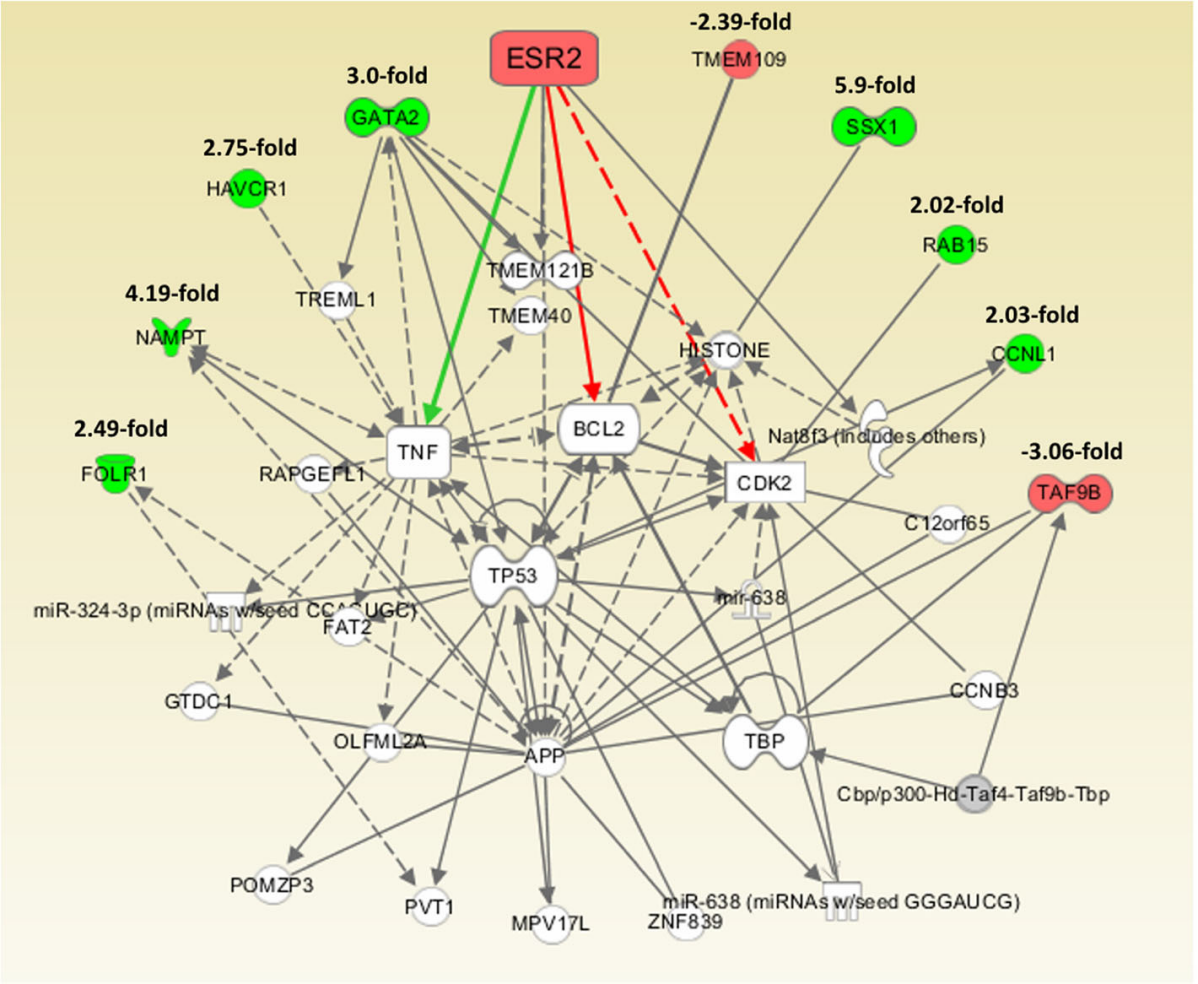
After knockdown of ERβ in HEC-1A cells, mRNA isolation and Affymetrix GeneChip transcriptome analysis, the resulting expression data was analyzed by Ingenuity IPA Software (Ingenuity Systems, Stanford, USA) providing a network connecting the indicated regulated genes with ERβ. Arrows indicate effects on expression, phosphorylation or direct binding. Dotted arrows: regulation of gene expression only. Solid arrows indicate combined effects on expression, phosphorylation or direct binding

**Fig. 5:**
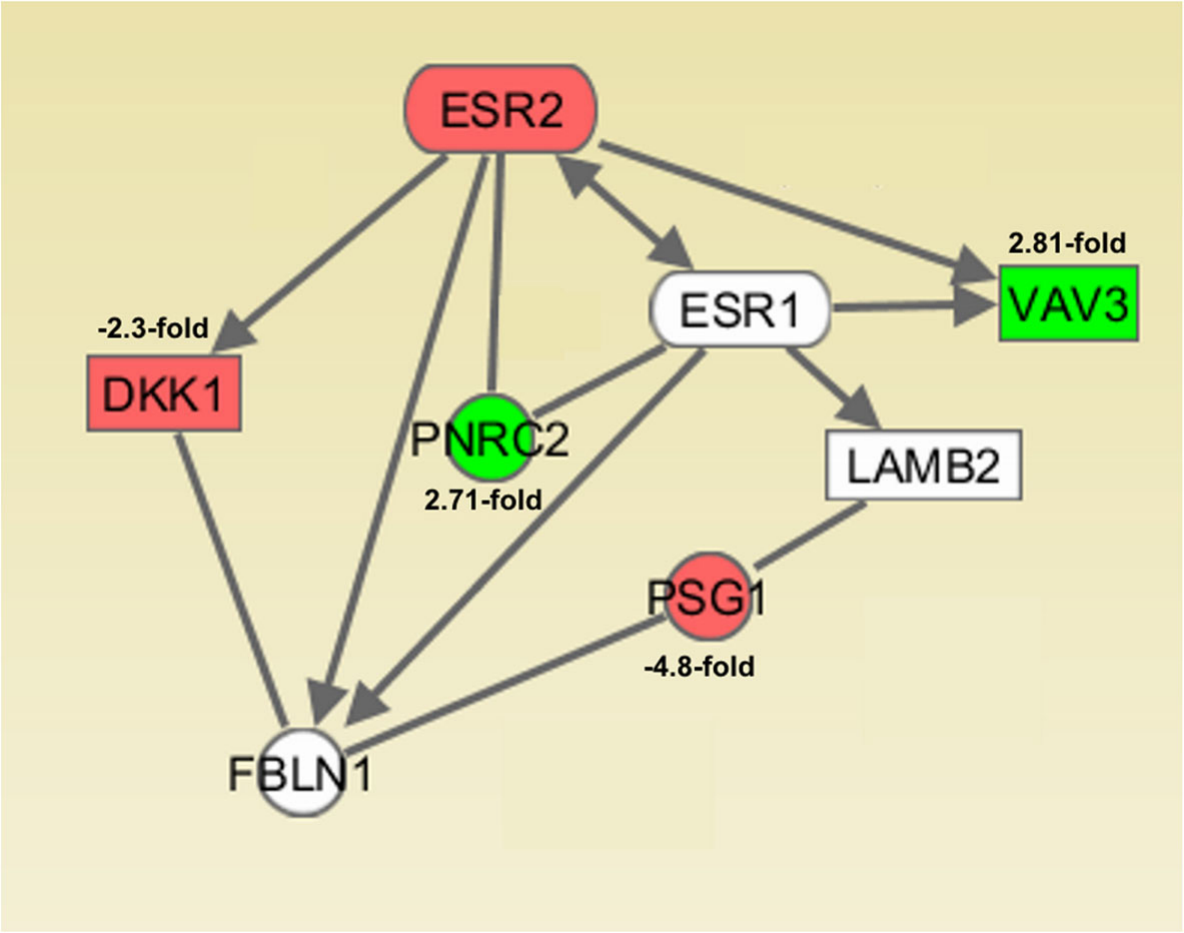
After knockdown of ERβ in RL95/2 cells, mRNA isolation and Affymetrix GeneChip transcriptome analysis, the results of Ingenuity IPA Software analysis (Ingenuity Systems, Stanford, USA) provide a network connecting the indicated regulated genes with ERα and ERβ. Solid arrows indicate combined effects on expression, phosphorylation or direct binding

After knockdown of ESR2 gene in HEC-1A and RL95/2 cells, we analyzed total RNA by means of Affymetrix Human 2.0 Gene Chips and applying a cut-off level of 2.0-fold change and a *p*-value < 0.05, we observed a set of 9 genes, 7 of which were up- and 2 were down-regulated on the mRNA level. In transfected RL95/2 cells, on the same way 6 differentially regulated genes were identified, two up- and 4 down-regulated ones (Table 1). Regulation of selected genes was confirmed by means of RT-qPCR (Fig. 3). Gene enrichment analysis by means of PANTHER Overrepresentation Test revealed annotated GO Ontology terms associated with the regulated genes (Additional file 2: Table S2) [22]. Finally, gene network analyses of the microarray data performed by Ingenuity IPA Software (Ingenuity Systems, Stanford, USA) suggested connections between ESR2 gene and the genes being regulated after its knockdown (Fig. 4, Fig. 5).

**Table 1:** Results from Affymetrix GeneChip 2.0 analysis: Genes with altered expression 72 h after knockdown of ESR2 gene in endometrial HEC-1A and RL95/2 cancer cells (cutoff value: 2.0, *p* < 0.05)

Gene symbol	Gene name	Regulation (−fold)
HEC-1A cells		
SSX1	Synovial sarcoma, X breakpoint 1	5,9
NAMPT	Nicotinamide phosphoribosyltransferase	4,19
GATA2	GATA binding protein 2	3,01
HAVCR1	Hepatitis A virus cellular receptor 1	2,75
FOLR1/ FR	Folate receptor 1	2,49
CCNL1	Cyclin L1	2,03
RAB15	Ras-related protein Rab-15	2,02
TMEM109	Transmembrane protein 109	-2,39
TAF9B	Transcription initiation factor TFIID subunit 9B	-3,06
RL95/2 cells		
VAV3	Guanine nucleotide exchange factor VAV3	2,81
PNRC2	Proline-rich nuclear receptor coactivator 2	2,71
DKK1	Dickkopf-related protein 1	-2,30
XAGE3	X antigen family member 3)	-2,48
MUC15	Mucin 15	-3,03
PSG1	Pregnancy specific beta-1-glycoprotein 1	-4,83

### Effect of ERβ modulators on proliferation of endometrial adenocarcinoma cells

**Fig. 6:**
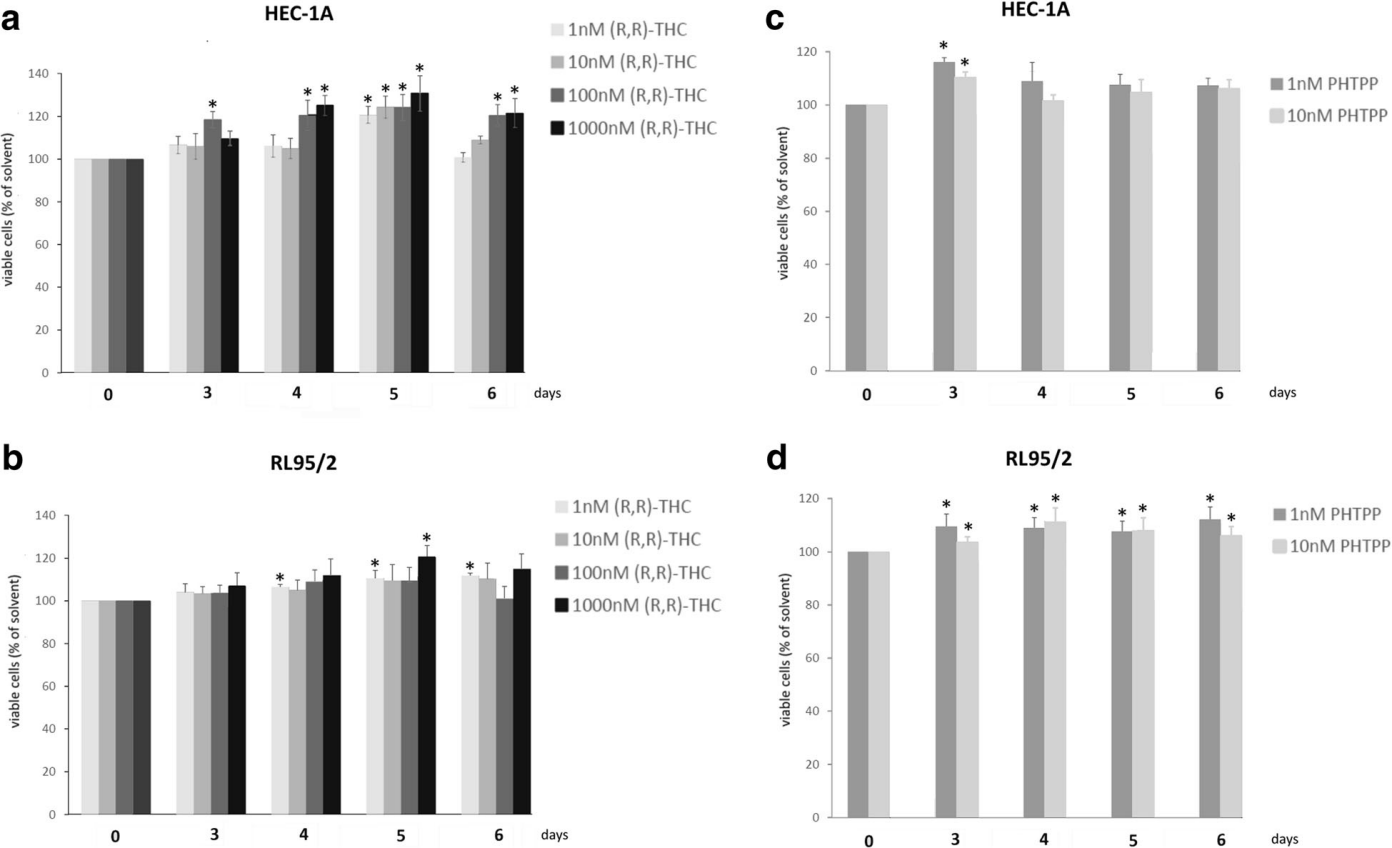
Effect of specific ERβ antagonists on proliferation of endometrial cancer cells. **a**) Effect of (R,R)-THC on HEC-1A cells, **b**) effect of (R,R)-THC on proliferation of RL95/2 cells, **c**) effect of PHTPP on HEC-1A cells, **d**) effect of PHTTP on RL95/2 cells. Antagonists were added in the indicated concentrations and cell growth was measured examining the relative numbers of viable cells in percent of day 0, using the CTB assay (PROMEGA). *p < 0.05 vs. solvent (*n* = 4)

To examine, to what extent inhibition of ERβ by specific antagonists would have similar effects on proliferation like knockdown of this receptor, we treated HEC-1A and RL95/2 cells with different concentrations of ERβ antagonists (R,R) THC and PHTPP (Tocris Bioscience). PHTPP is a pyrazolo[1,5-α]pyrimidine-based ligand that acts as a full antagonist of estrogen ERβ receptors with 36-fold selectivity over ERα. It exhibits no significant agonism on ERα or ERβ [23]. At 100 pM, PHTPP has been previously found to enhance SKOV3 and OV2008 ovarian cancer cell growth in in vitro assays [24]. (R,R) THC is the abbreviation for (R,R)-5,11-Diethyl-5,6,11,12-tetrahydro− 2,8-chrysenediol, which is a non-steroidal, selective estrogen receptor ligand and antagonist at ERβ receptor (Ki = 3.6 nM) [25]. Treatment with (R,R) THC (1 to 1000 nM) resulted in a significant increase of proliferation of HEC-1A cells in a dose-dependent manner. Maximum effects were observed after 5 days of treatment, with an increase by 21 ± 3.9% (1 nM) (*p* < 0.01), by 24 ± 5.1% (10 nM) (p < 0.01), by 24 ± 6.2% (100 nM) (p < 0.01) and by 28 ± 8% (1000 nM) (p < 0.01) (Fig. 6a). In contrast, the effects of (R,R) THC on RL95/2 cells were smaller, with maximum increases of proliferation by 9 ± 2.4% (1 nM) and by 12 ± 2.5% (1000 nM) after 5 days of treatment (both *p* < 0.05) (Fig. 6b). Treatment with 1 and 10 nM PHTTP increased proliferation of HEC-1A cells by 16 ± 2.1% or 10 ± 1.5%, respectively (both *p* < 0.01) 3 days after treatment (Fig. 6c). Smaller, but statistically significant effects were observed after PHTPP treatment of RL95/2 cells, which began at day 3 of treatment and lasted until the end of the test period (6 days). The maximum proliferation increase was 15 ± 1.9% on day 6 (*p* < 0.05) triggered by 1 nM PHTPP (Fig. 6d).

Treatment with the ERβ agonists ERB-041 and Liquiritigenin in turn did not lead to growth inhibition, only the agonist WAY200070 slightly decreased cell proliferation of HEC-1A cells with a maximum by 12 ± 3,8% (10 nM, day 7) and of RL95/2 cells by up to 7 ± 1.4% (10 nM) (both *p* < 0.05) (data not shown).

## Discussion

In this study, we knocked down expression of ERβ in two endometrial cancer cell lines and observed enhanced proliferation both of ERα-positive RL95/2 and ERα-negative HEC-1A cells. Transcriptome analysis revealed that this was accompanied by increased expression of several genes being known to be upregulated in cancer, including proliferation-associated genes and oncogenes, and by repression of genes associated with differentiation, apoptosis or growth inhibition. Though the transcriptome of both cell lines was affected differentially, our data suggest that ERβ might have tumor suppressing features in endometrium which can be both ERα-dependent and -independent. Our data are in line with previous studies reporting that knockdown of ERβ led to increased proliferation of cancer cells primarily of the breast, prostate or ovary, whereas overexpression of this receptor inhibited proliferation [14, 26–29]. In a previous study, we were able to show, that expression of ERβ was even sufficient to significantly inhibit proliferation of hormone-independent COS-1 cells and to increase apoptosis even in the absence of E2 [30]. However, the role of this receptor in endometrial cancer is still controversial, as some studies reported downregulation of ERβ in endometrial cancer [7–9], and others observed increased expression of this receptor in endometrial tumor tissue or its association with disease progression [10–12]. Concordant studies on other cancer entities demonstrated that ERβ expression is decreased or lost in a variety of tumors when compared to normal tissue, a fact that was reported to have negative consequences on survival or therapy of different cancer entities [31–35]. Considering all these results, previously a role of ERβ as putative tumor suppressor has been suggested for a variety of cancer entities like prostate cancer, breast cancer, ovarian cancer or malignant pleural mesothelioma [36, 37]. The data of our study suggest that this tumor suppressor feature of ERβ might also be present in endometrial cancer.

In addition to siRNA-triggered ERβ knockdown we tested the effect of specific ERβ-agonists and -antagonists on endometrial cancer cell proliferation. Our observation, that several concentrations of the used antagonists were able to at least slightly enhance proliferation of both endometrial cancer cell lines, corroborates the results obtained from the knockdown experiments. The fact that these effects were smaller than expected might be explained by the known ligand-independent action of ERβ [38]. This action might also underlie the lacking anti-proliferative effect of its agonists ERB-041 and Liquiritigenin. However, treatment with ERβ agonist WAY200070 was able to slightly decrease growth of both cell lines, which further supports the growth-inhibitory role of this receptor in endometrial cancer cells.

With regard to the transcriptome analysis we performed to elucidate the effect of an ERβ knockdown on gene expression of endometrial cancer cells, a different set of genes was regulated in both cell lines, possibly due to their different ERα status. Gene enrichment analysis revealed annotated GO-terms with regard to biological processes, which in part might explain the results of our proliferation experiments. For example, genes down-regulated after ESR2 knockdown in RL95/2 cells were associated with the GO term “negative regulation of canonical Wnt signaling pathway (GO:0090090)”. As the Wnt pathway is known to promote proliferation of tumor cells [39], its up-regulation might be one reason for the increased growth of this cell line transfected with ESR2 siRNA. Gene network analyses of the microarray data performed by Ingenuity IPA Software (Ingenuity Systems, Stanford, USA) suggested a connection between ESR2 gene and the genes being regulated after its knockdown. With regard to HEC-1A cells, a network was elucidated in which most of these interactions seem to be mediated by central key molecules like TNF, BCL2, CDK2 and p53 (Fig. 4). However, it is remarkably, that in this network both ESR2 and all genes regulated after its knockdown could be connected at least illustrating some of the mechanisms underlying the observed transcriptome change in this cell line. In RL-95/2 cells, Ingenuity IPA analysis was not able to connect all regulated genes to ESR2, but the ERα expressed in this cell line, activated by diminished expression of his antagonist ERβ, seems to be the most important mediator changing gene expression leading to upregulation of VAV3 and PNRC2 and downregulation of DKK1 and PSG1 (Fig. 5).

To discuss the potential role of the regulated genes in the proliferation increase we observed in both endometrial cancer cell lines, first, in HEC-1A cells, after knockdown of ERβ, expression of cyclin L1 (CCNL1) was found to be induced 2-fold, which is tempting to speculate as one mechanism underlying the observed proliferation increase. Indeed, CCNL1 has been reported to activate not only cyclin-dependent protein serin/threonine kinases, but also phosphorylation and transcription activity of RNA polymerase II. With regard to cancer, CCNL1 has been reported to be overexpressed in head and neck squamous cell carcinomas and thus is considered as a candidate proto-oncogene [40, 41]. The gene exhibiting the strongest induction after ESR2-knockdown was SSX-1, a transcription factor with elusive oncogenic functions expressed in a variety of human tumors of epithelial and mesenchymal origin [42]. Though originally being a transcriptional repressor, this protein is known to interact with other molecules like SS18, thereby deregulating developmental programs to drive transformation leading to irreversible mesenchymal differentiation [43]. The 2-fold upregulated gene RAB15 is known as a member of RAS oncogene family, but to judge, to what extent it acts as an oncogene itself or might be able to affect proliferation of cancer cells, more research on its specific functions is necessary [44, 45]. Nicotinamide phosphoribosyltransferase (NAMPT), in our experiments more than 4-fold upregulated after ESR2 knockdown in HEC-1A cells, is overexpressed in several cancer entities such as ovarian, breast, gastric, colorectal, and prostate cancer, gliomas and B-cell lymphomas [46]. Nicotinamide adenine dinucleotide (NAD) is rapidly turned over by cancer cells, but they do not efficiently utilize the de novo synthesis pathway. Thus, they are more dependent on NAD regeneration by the NAMPT pathway, which makes this enzyme a potential target for cancer therapy [47]. A number of selective NAMPT small molecule inhibitors have been demonstrated to exert considerable anti-tumor activity in in vitro and in vivo tumor models. Thus, the observed overexpression of NAMPT is expected to provide more NAD und thus could be another explanation of the observed growth increase. GATA2, 3-fold overexpressed after ESR2 knockdown in HEC-1A cells, is a transcription factor, which has been reported to be overexpressed in non-familial EVI1-positive acute myeloid leukemia as well as in prostate cancer. In both cancer types, its overexpression is associated with cancer progression, aggressiveness and an adverse prognosis for patients survival [48, 49]. On the cellular level, GATA2 overexpression in prostate cancer cells increases their proliferation, motility and invasiveness [50]. To which extent similar actions of GATA2 might explain the observed enhanced proliferation of HEC-1A endometrial cancer cells, has to be investigated. The most strongly down-regulated gene in HEC-1A cells transfected with ESR2 siRNA is apoptosis gene TAF9B. In a recent study, among others, loss of heterozygosity of TAF9B has been identified to be associated with metastasis-free survival in breast cancer patients, indicating its potential value as prognostic marker [51]. Expression of TAF9b further has been described to be essential for cell viability, as it has a key role in transcription initiation of RNA polymerase II preinitiation complex assembly [52]. Importantly, TAF9B recently has also been identified to be an transcription coactivator for tumor suppressor p53 [53]. Thus, it has to be examined in further studies, to what extent weakening of p53 action through TAF9B downregulation after ESR2 knockdown is able to increase cell proliferation as we observed in HEC-1A cells.

In ERα/β-positive RL95/2 cells, knockdown of ESR2 expression led to dysregulation of another set of genes, which might be partially explained by its positive ERα status. Guanine nucleotide exchange factor VAV3, which was 2.81-fold elevated after ESR2 knockdown, has been reported to be an ER-coactivator and oncogene and to be overexpressed in endometrial cancer [54]. A recent study reported that inhibition of VAV3 by a specific miRNA was able to reduce proliferation and metastasis of non-small lung cancer cells [55].Taken together, it seems plausible that overexpression of VAV3 might have contributed to the enhanced proliferation we observed in transfected RL95/2 cells. The polyproline-rich nuclear receptor coactivator PNRC2, which was 2.71-fold elevated after ESR2 knockdown, is also known to be an ERα-coactivator [56]. Thus, it is tempting to speculate that enhanced ERα activity (less limited by ERβ after transfection) in RL95/2 cells might also have contributed to the increased proliferation of this line. The gene which was most significantly down-regulated after ESR2 knockdown in RL95/2 cells was pregnancy specific glycoprotein PSG1, a member of the carcinoembryonic antigen (CEA) gene family. As PSG1 is considered as a marker for endometrial differentiation, its decrease might be a sign of further RL95/2 cell de-differentiation triggered by loss of ERβ [57]. Finally, knockdown of ERβ resulted in more than 2-fold decrease of DKK1 expression, which is an important inhibitor of the Wnt signaling pathway playing an essential role in tumor invasion and migration. In a recent study, knockdown of DKK1 in endometrial Ishikawa cancer cells led to enhanced proliferation, migration and invasion [58]. Thus, DKK1 downregulation in our setting is another molecular mechanism which might underlie the enhanced proliferation of RL95/2 cells transfected with ERβ siRNA.

## Conclusions

In conclusion, our data show that expression of ERβ plays an important role in proliferation of two endometrial cancer cell lines, which is mediated by transcriptome changes being judged to be plausible to underlie this effect. Our data suggest that ERβ is able to suppress cancer-associated genes, partially involved in proliferation control, and to activate expression of genes maintaining cellular differentiation, apoptosis and growth inhibition. Though the data of this in vitro *study* need to be verified in the in vivo situation, they suggest that ERβ might act as a tumor suppressor in endometrium and encourage further studies to what extent this receptor might be a putative therapy target in this cancer entity.

## Additional files

Additional file 1: **Table S1** Primers used for RT-qPCR analyses. (DOCX 14 kb)

Additional file 2: **Table S2** Gene enrichment analysis of genes significantly regulated after knockdown of ESR2 in HEC-1A and RL95/2 cells based on the microarray results. Analysis Type: PANTHER Overrepresentation Test (Released 20190429). Annotation Version and Release Date: GO Ontology database Released 2019-02-02. Shown are the top 10 significant biological processes. Test type: Fisher’s exact test with Bonferroni correction. (DOCX 32 kb)

## Data Availability

The datasets used and/or analysed during the current study are available from the corresponding author on reasonable request. Affymetrix microarray data are available as Excel files. NCBI accession numbers are not available due to a damaged CEL file.

## Abbreviations

(R,R) THC: (R,R)-5,11-Diethyl-5,6,11,12-tetrahydro− 2,8-chrysenediol
CTB: Cell Titer Blue
ER: estrogen receptor
NAD: nicotinamide adenine dinucleotide
PHTPP: 4-[2-Phenyl-5,7-bis(trifluoromethyl)pyrazolo[1,5-a]pyrimidin− 3-yl]phenol
