# Supplementary material for: Knockdown of estrogen receptor β increases proliferation and affects the transcriptome of endometrial adenocarcinoma cells
Source: BMC Cancer. 2019 Jul 29;19:745. doi: 10.1186/s12885-019-5928-2 (PMC6664594; doi:10.1186/s12885-019-5928-2)
Supplement: Supplementary file 1 — Table S1 Primers used for RT-qPCR analyses. (DOCX 14 kb) [file 12885_2019_5928_MOESM1_ESM.docx]

**Additional file 1: Table S1.** Primers used for RT-qPCR analyses.

| **Gene** | **PCR primers (5´-3´)** | **Amplicon (bp)** |
| --- | --- | --- |
| ESR2 | GGGAGCCCTCTTTGCTTTT  GGCATGCGAGTAACAAGGGC | 177 |
| ACTB | CTTCCTTCCTGGGCATGGAGT  CAGGAGGAGCAATGATCTTGATCTTC | 210 |
| NAMPT | CTTCGGTTCTGGTGGAGGTT  ATCGGCCCTTTTTGGACCTT | 152 |
| CCNL1 | AACCCTGGTTCAAACTGCCT  GGCAACGGAATCTGAAGTGC | 132 |
| DKK1 | GTGCAAATCTGTCTCGCCTG  ACCAAAGCTTTCAGTGATGGT | 156 |
| VAV3 | CTGCCAGCTGCTTAACAACC  CAGGCCGTGAGAAATGTCCT | 117 |
