# Supplementary material for: Knockdown of estrogen receptor β increases proliferation and affects the transcriptome of endometrial adenocarcinoma cells
Source: BMC Cancer. 2019 Jul 29;19:745. doi: 10.1186/s12885-019-5928-2 (PMC6664594; doi:10.1186/s12885-019-5928-2)
Supplement: Supplementary file 2 — Table S2 Gene enrichment analysis of genes significantly regulated after knockdown of ESR2 in HEC-1A and RL95/2 cells based on the microarray results. Analysis Type: PANTHER Overrepresentation Test (Released 20190429). Annotation Version and Release Date: GO Ontology database Released 2019-02-02. Shown are the top 10 significant biological processes. Test type: Fisher’s exact test with Bonferroni correction. (DOCX 32 kb) [file 12885_2019_5928_MOESM2_ESM.docx]

**Additional file 2: Table S2.** Gene enrichment analysis of genes significantly regulated after knockdown of ESR2 in HEC-1A and RL95/2 cells based on the microarray results. Analysis Type: PANTHER Overrepresentation Test (Released 20190429). Annotation Version and Release Date: GO Ontology database Released 2019-02-02. Shown are the top 10 significant biological processes. Test type: Fisher´s exact test with Bonferroni correction [1].

| **Genes up-regulated after ESR2 knockdown in HEC-1A cells** | | |
| --- | --- | --- |
| **GO biological processes** | **Fold enrichment** | **p-value** |
| cellular process involved in reproduction in multicellular organism (GO:0022412) | 61.21 | 1.85E-02 |
| multicellular organism reproduction (GO:0032504) | 53.56 | 2.08E-02 |
| phosphorylation of RNA polymerase II C-terminal domain (GO:0070816) | 42.85 | 2.53E-02 |
| cellular response to light stimulus (GO:0071482) | 32.96 | 3.21E-02 |
| positive regulation of JNK cascade (GO:0046330) | 30.61 | 3.44E-02 |
| single fertilization (GO:0007338) | 30.61 | 3.44E-02 |
| interleukin-6 production (GO:0032635) | 28.57 | 3.66E-02 |
| regulation of stress-activated MAPK cascade (GO:0032872) | 26.78 | 3.89E-02 |
| regulation of JNK cascade (GO:0046328) | 26.78 | 3.89E-02 |
| regulation of microtubule polymerization or depolymerization (GO:0031110) | 25.21 | 4.11E-02 |
| **Genes down-regulated after ESR2 knockdown in HEC-1A cells** | | |
| **GO biological processes** | **Fold enrichment** | **p-value** |
| microtubule polymerization or depolymerization (GO:0031109) | > 100 | 1.25E-02 |
| membrane protein proteolysis (GO:0033619) | 42.42 | 1.28E-03 |
| iron ion import (GO:0097286) | 39.77 | 2.79E-02 |
| intrinsic apoptotic signaling pathway in response to DNA damage (GO:0008630) | 28.92 | 3.70E-02 |
| negative regulation of cell death (GO:0060548) | 26.51 | 4.00E-02 |
| histone H3 acetylation (GO:0043966) | 26.51 | 4.00E-02 |
| macromolecule modification (GO:0043412) | 24.47 | 4.30E-02 |
| phospholipid biosynthetic process (GO:0008654) | 21.21 | 4.90E-02 |
| cell-matrix adhesion (GO:0007160) | 12.72 | 1.16E-02 |
| response to nitrogen compound (GO:1901698) | 12.24 | 1.25E-02 |
| **Genes up-regulated after ESR2 knockdown in RL95/2 cells** | | |
| **GO biological processes** | **Fold enrichment** | **p-value** |
| organic cation transport (GO:0015695) | 68.61 | 1.61E-02 |
| cyclic nucleotide metabolic process (GO:0009187) | 32.5 | 3.19E-02 |
| positive regulation of adaptive immune response (GO:0002821) | 29.41 | 3.50E-02 |
| positive regulation of immune response (GO:0050778) | 28.07 | 3.65E-02 |
| positive regulation of immune system process (GO:0002684) | 23.75 | 4.28E-02 |
| nucleosome assembly (GO:0006334) | 21.29 | 4.74E-02 |
| regulation of cell communication (GO:0010646) | 5.16 | 2.04E-02 |
| regulation of signal transduction (GO:0009966) | 5.16 | 2.04E-02 |
| cell surface receptor signaling pathway (GO:0007166) | 3.45 | 2.80E-02 |
| **Genes down-regulated after ESR2 knockdown in RL95/2 cells** | | |
| **GO biological processes** | **Fold enrichment** | **p-value** |
| negative regulation of protein modification process (GO:0031400) | > 100 | 1.14E-02 |
| negative regulation of cellular protein metabolic process (GO:0032269) | 87.48 | 1.32E-02 |
| hormone biosynthetic process (GO:0042446) | 74.99 | 1.51E-02 |
| steroid biosynthetic process (GO:0006694) | 58.32 | 1.89E-02 |
| response to endoplasmic reticulum stress (GO:0034976) | 34.99 | 3.00E-02 |
| cholesterol metabolic process (GO:0008203) | 27.63 | 3.74E-02 |
| regulation of secretion by cell (GO:1903530) | 26.24 | 3.92E-02 |
| negative regulation of canonical Wnt signaling pathway (GO:0090090) | 21 | 4.83E-02 |
| neurotransmitter secretion (GO:0007269) | 15.91 | 7.42E-03 |
| immune system process (GO:0002376) | 5.08 | 1.10E-03 |

References

1. Mi H, Muruganujan A, Huang X, Ebert D, Mills C, Guo X, Thomas PD. Protocol Update for large-scale genome and gene function analysis with the PANTHER classification system (v.14.0). Nat Protoc. 2019;14:703–21. doi:10.1038/s41596-019-0128-8.
